# Icteric Leptospirosis Leading to Multiorgan Failure and Concomitant Pancreatitis

**DOI:** 10.7759/cureus.38350

**Published:** 2023-04-30

**Authors:** Justin Canakis, Michael Bechara, Nouf Turki, Francis Carro Cruz, Jaclyn E Kagihara, Marie L Borum, Samuel A Schueler

**Affiliations:** 1 Internal Medicine, George Washington University School of Medicine and Health Sciences, Washington DC, USA; 2 Gastroenterology and Hepatology, The George Washington University School of Medicine and Health Sciences, Washington DC, USA; 3 Gastroenterology and Hepatology, George Washington University School of Medicine and Health Sciences, Washington DC, USA

**Keywords:** infectious disease epidemiology, pancreatitis, acute kidney injury, acute liver failure, leptospirosis

## Abstract

Leptospirosis is a zoonotic disease caused by the spirochete *Leptospira interrogans* with a majority of cases occurring in the tropics. Diagnosing leptospirosis is challenging due to the variable and non-specific clinical presentation. While severe leptospirosis may present with renal failure, liver failure, and pulmonary hemorrhage, there are few described cases of renal failure and liver failure accompanied by pancreatitis and dysrhythmias, particularly in temperate climates.

We present a case of severe leptospirosis presenting with bilateral calf pain, acute oliguric renal failure, acute liver failure, dysrhythmias, and pancreatitis. Clinicians must consider this diagnosis in temperate climates and consider testing and empirically treating for leptospirosis in patients with similar symptom constellations, vague symptoms, and lab abnormalities of unknown etiology.

## Introduction

Leptospirosis is a zoonotic disease caused by the spirochete *Leptospira interrogans*. Leptospirosis is considered to be the most widespread zoonotic disease in the world with an estimated one million cases each year resulting in 59,000 deaths [1]. Leptospirosis exists in diverse epidemiologic settings, but the incidence of this disease is significantly higher in tropical and subtropical regions [2]. In 2019, the United States and its territories reported 189 cases of leptospirosis with 93 and 30 cases occurring in Puerto Rico and Hawaii, respectively [3]. Leptospires spread in the urine of animal hosts and can survive in water and wet soil for weeks to months [4]. Humans can be infected when urine or contaminated water enters the body via ingestion, breaches in the skin, or contact with mucous membranes [4]. Risk factors for leptospirosis include occupational exposure for people who work outdoors or with animals, indulge in water sports, such as surfing or kayaking, and have unsanitary living conditions.

There are two forms of leptospirosis, anicteric and icteric. The anicteric form is self-limited and presents with non-specific flu-like symptoms and represents 90% of cases [5]. The clinical presentation of icteric leptospirosis can include multiorgan failure with complications, including aseptic meningitis, renal failure, liver failure, pulmonary hemorrhage, acute respiratory distress syndrome, dysrhythmias, and uveitis [6]. Cases of pancreatitis are rare [7]. We present a unique case of leptospirosis causing acute renal failure, acute liver failure, dysrhythmias, and pancreatitis. This article was previously presented as a meeting abstract at the 2022 American College of Gastroenterology Conference in October 2022.

## Case presentation

A 36-year-old male with a history of schizoaffective disorder on olanzapine and polysubstance use disorder presented with bilateral calf pain and pruritis of one-week duration. The patient reported taking naproxen 1,000 milligrams (mg) daily and ibuprofen 400 mg daily for one week for his calf pain. The patient also endorsed smoking phencyclidine (PCP) and smoking marijuana one week prior to presentation. He also reported that he drinks one pint of liquor weekly, but denied any recent use because he could not afford it. He denied recent travel, contact with animals, excessive exercise, or prolonged immobilization.

**Figure 1:**
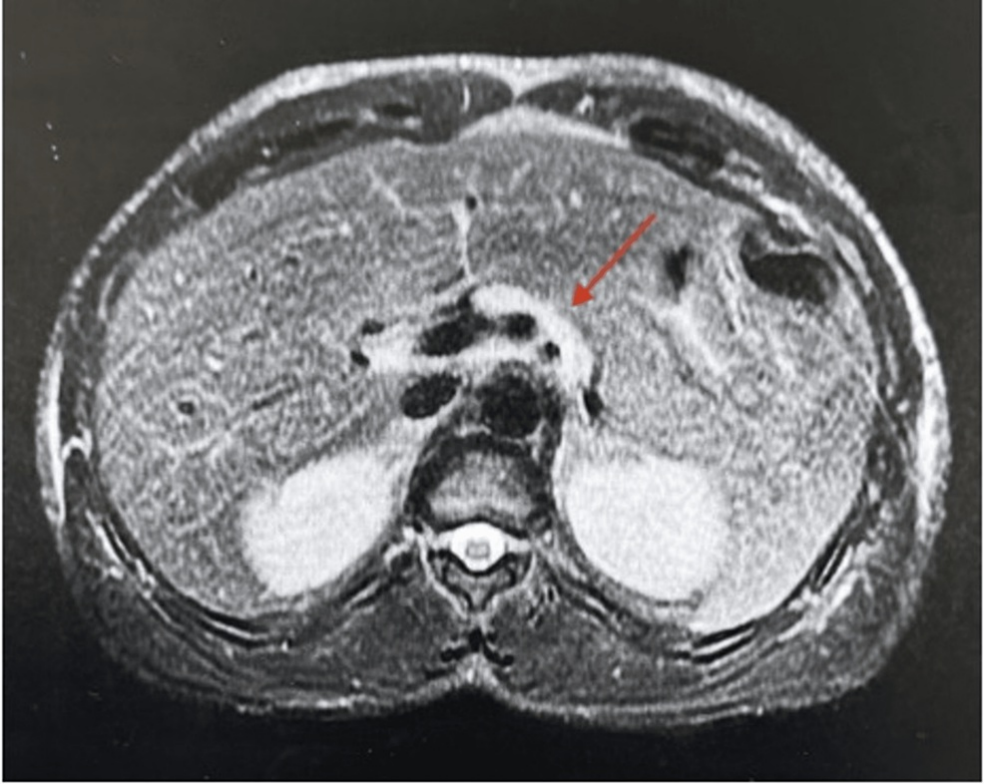
T2 Fat-saturated image showing high /bright signal edema surrounding the head of the pancreas

**Figure 2:**
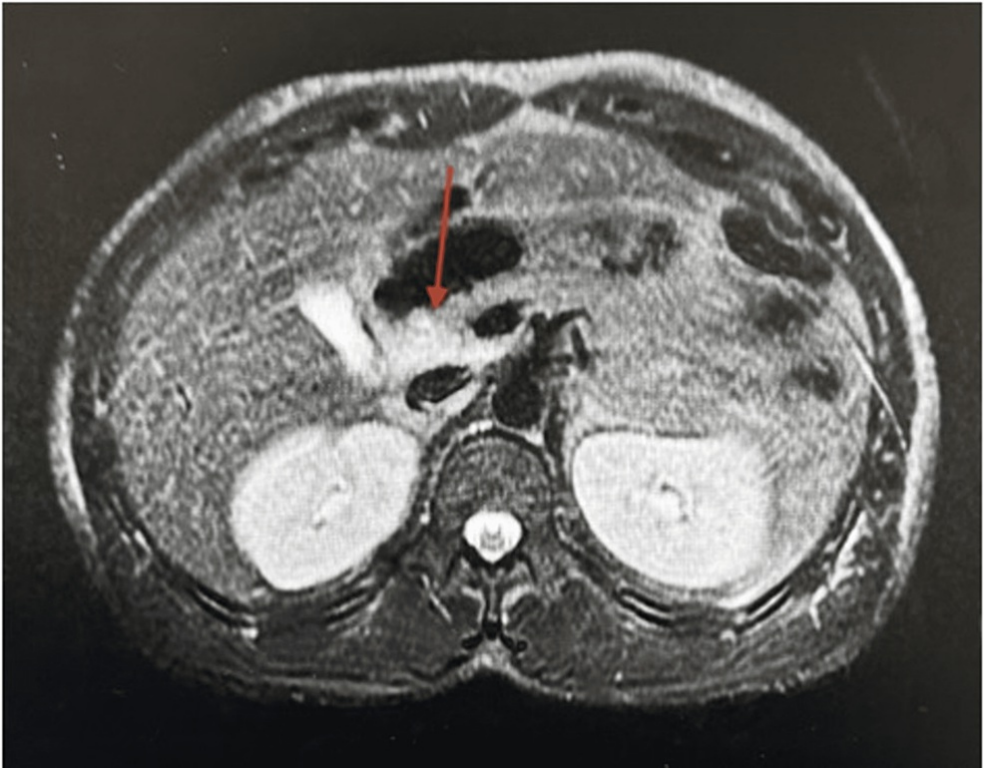
T2 Fat-saturated image showing high/bright signal edema surrounding the body of the pancreas

Vital signs were normal except for a heart rate of 118 beats per minute. The exam revealed scleral icterus and bilateral calf tenderness to palpation. Laboratory evaluation was pertinent for a white blood cell count of 12,750 per microliter thousand per milliliter (ml), platelet count of 57,000 per ml, blood urea nitrogen (BUN) of 91 milligrams/deciliter (mg/dl), creatinine of 7.8 mg/dl, aspartate aminotransferase (AST) of 392 units/liter (U/L), alanine aminotransferase (ALT) of 134 U/L, albumin of 3.1 grams/dl, alkaline phosphatase of 105 U/L, total bilirubin of 14.6 mg/dl, direct bilirubin of 9.1 mg/dl, international normalized ratio (INR) of 1.15, lipase of 912 U/L, and creatinine kinase of 7,000 U/L. Acetaminophen level was undetectable, and broad infectious workup, including a viral hepatitis panel, was negative. A phosphatidylethanol (PEth) level was not drawn to confirm any recent alcohol use. Bilateral lower extremity ultrasounds were negative for deep vein thrombosis (DVT). A right upper quadrant ultrasound revealed focal fatty infiltration in the right hepatic lobe. Magnetic resonance cholangiopancreatography (MRCP) revealed mild fatty infiltration without intrahepatic or extrahepatic bile duct dilatation and mild edema around the pancreatic head and neck consistent with pancreatitis (Figures 1, 2).

The patient was initially treated with supportive measures, including fluid resuscitation. He developed pleuritic chest pain. The electrocardiogram showed ST elevations in the lateral leads. Troponin was mildly elevated to 0.059 nanograms (ng)/ml, and B-type natriuretic peptide was elevated to 38,400 picograms (pg)/ml. A transthoracic echocardiogram revealed an ejection fraction reduced to 36% from 40-45% on admission. He then developed anuria and hypotension. He was transferred to the intensive care unit for intravenous fluids, vasopressors, steroids, and intermittent hemodialysis. His hyperbilirubinemia worsened and peaked at 40 mg/dl. Given the patient's vague clinical symptoms and multiorgan failure, leptospirosis antigen and polymerase chain reaction (PCR) were obtained, and ceftriaxone 2g IV for 10 days was empirically started. He began to improve with normalization of blood pressure, urine output, bilirubin levels, transaminases, and renal function, and he was discharged. The results for leptospirosis antigen and PCR were positive. He was seen three months later in the emergency department for a head laceration, but no labs were drawn. To date, the patient has been lost to follow-up.

## Discussion

Leptospirosis is an emerging infectious disease with variable clinical presentation. Initial symptoms include sudden-onset fevers, chills, headache, severe myalgias, conjunctival suffusion, anorexia, nausea, vomiting, and prostration [6]. Our working hypothesis on how the patient was infected with leptospirosis is from rat urine, given that he was experiencing homelessness and living on the streets. Notably, our patient’s chief complaint was a sudden onset of bilateral calf tenderness; muscle tenderness in the calves can be a distinguishing physical finding in the acute phase [6]. Severe leptospirosis can lead to dysfunction in multiple organs, including the kidneys, liver, lungs, brain, and heart. Our patient presented with many classic signs of severe leptospirosis, including renal, hepatic, and cardiac dysfunction. Interestingly, our patient also had pancreatitis, a rare manifestation of leptospirosis. While the patient did endorse alcohol use, he denied a history of heavy alcohol use or prior pancreatitis. The mechanism of acute pancreatitis in leptospirosis is incompletely understood. It has been proposed that activation of toll-like receptor 2 may trigger an inflammatory pathway leading to vasculitis and endothelial damage to the pancreas [8]. A literature review conducted by Maier et al. (2019) found that only 17 cases of leptospirosis-associated pancreatitis have been published in the literature from 2002-2019 and that they were mainly localized to Central Europe and Sri Lanka [7]. Furthermore, only one such case of leptospirosis-associated pancreatitis has been documented in North America [9]. The patient in the aforementioned case was experiencing homelessness and presented with abdominal pain. He was found to have elevated lipase and abdominal pain, but his MRCP was unremarkable for pancreatitis [9]. He also developed acute renal failure requiring dialysis [9].

## Conclusions

Leptospirosis is the most widespread zoonotic disease in the world and can exist in diverse epidemiologic settings, including urban environments. Clinicians should be aware of the risk factors and presentation of leptospirosis, particularly in severe cases, to avoid patient morbidity and mortality.

## References

[1] (2023). Leptospirosis. Information for health care workers. https://www.cdc.gov/leptospirosis/health_care_workers/.

[2] Mazhar M, Kao JJ, Bolger DT Jr (2016). A 23-year-old man with leptospirosis and acute abdominal pain. Hawaii J Med Public Health.

[3] TABLE 2i (2023). 2019 annual tables of infectious disease data. https://www.cdc.gov/nndss/data-statistics/infectious-tables/index.html.

[4] (2023). Infection. https://www.cdc.gov/leptospirosis/infection/index.html.

[5] Wang S, Stobart Gallagher MA, Dunn N (2023). Leptospirosis. https://www.ncbi.nlm.nih.gov/books/NBK441858/.

[6] Bharti AR, Nally JE, Ricaldi JN (2003). Leptospirosis: a zoonotic disease of global importance. Lancet Infect Dis.

[7] Maier A, Kaeser R, Thimme R, Boettler T (2019). Acute pancreatitis and vasoplegic shock associated with leptospirosis - a case report and review of the literature. BMC Infect Dis.

[8] Gomes PE, Brilhante SO, Carvalho RB, Sousa DR, Daher EF (2019). Pancreatitis as a severe complication of leptospirosis with fatal outcome: a case report. Rev Inst Med Trop Sao Paulo.

[9] Afzal I, Thaker R, Weissman S, Kothari M (2020). Leptospirosis as an unusual culprit of acute pancreatitis and portal vein thrombosis in a New Yorker. Clin Case Rep.

